# Visceral Leishmaniasis: A Differential Diagnosis to Remember after Bone Marrow Transplantation

**DOI:** 10.1155/2014/587912

**Published:** 2014-12-11

**Authors:** Margarida Dantas Brito, Fernando Campilho, Rosa Branca, Carlos Pinho Vaz, Cristina Silva, Teresa Sousa, Carlos Mendes, António Campos

**Affiliations:** ^1^Bone Marrow Transplantation Service, Instituto Português de Oncologia Francisco Gentil, Rua Dr. António Bernardino de Almeida, 4200-072 Porto, Portugal; ^2^Laboratory of Hematology, Instituto Português de Oncologia Francisco Gentil, Rua Dr. António Bernardino de Almeida, 4200-072 Porto, Portugal

## Abstract

Leishmania infection in immunocompromised hosts is reported in the literature, mostly concerning human immunodeficiency virus infected patients. It is not well characterized in the context of stem cell transplantation. We report a rare case clinical case of visceral leishmaniasis after allogeneic bone marrow transplantation. A 50-year-old Caucasian male was referred to allogeneic bone marrow transplantation with a high-risk acute lymphoblastic B leukemia in first complete remission. Allogeneic SCT was performed with peripheral blood stem cells from an unrelated Portuguese matched donor. In the following months, patient developed mild fluctuating cytopenias, mostly thrombocytopenia (between 60 and 80∗10^9^/L). The only significant complaint was intermittent tiredness. The common causes for thrombocytopenia in this setting were excluded—no evidence of graft versus host disease, no signs of viral or bacterial infection, and no signs of relapsed disease/dysplastic changes. The bone marrow smear performed 12 months after transplantation revealed an unsuspected diagnosis: a massive bone marrow infiltration with amastigotes.

## 1. Introduction

Leishmaniasis is an emerging infectious disease, with increasing reports in endemic and nonendemic countries [1]. Additionally, in the past years, some case reports of leishmania infection in immunocompromised hosts appeared in the literature. Most reports refer to HIV infected patients or clinical cases in the setting of solid organ transplantation. There are scarse reports in the context of stem cell transplantation (SCT) [2–4]. The aim of this case report is to present a case of visceral leishmaniasis after allogeneic SCT.

## 2. Case Presentation

**Figure 1:**
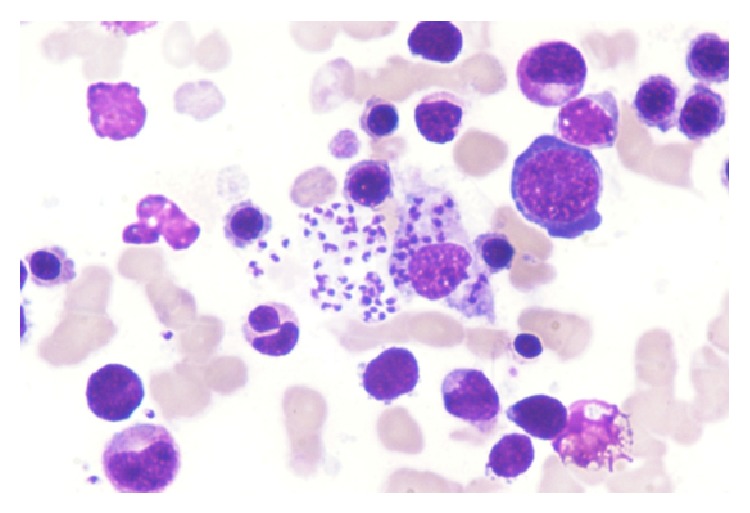
Photography of bone marrow smear showing massive bone marrow infiltration with amastigotes. Optical microscopy, Wright Giemsa stain, 100x magnification.

A 50-year-old Caucasian male, with no relevant past medical records, was referred to SCT with a high-risk acute lymphoblastic B leukemia in first complete remission (CR). Allogeneic SCT was performed with peripheral blood stem cells from an unrelated Portuguese matched 9/10 donor (disparity in locus HLA-A), after myeloablative conditioning regimen (with busulfan, cyclophosphamide, and antilymphocytic serum). Graft versus host disease prophylaxis was performed with tacrolimus and methotrexate. There were no major complications during neutropenic period. Corticotherapy was prescribed for upper digestive graft versus host disease diagnosed 2 months after SCT. In the following months, patient developed mild fluctuating cytopenias, mostly thrombocytopenia (between 60 and 80∗10^9^/L). The only significant complaint was intermittent tiredness. Bone marrow evaluation had no signs of relapse disease and patient had complete chimerism. The common causes for thrombocytopenia were excluded—no evidence of graft versus host disease; no signs of viral or bacterial infection; and no signs of relapsed disease/dysplastic changes. The bone marrow smear performed 12 months after transplant revealed an unsuspected diagnosis: a massive bone marrow infiltration with amastigotes—(Figure 1 optical microscopy, Wright Giemsa stain, 100x magnification). Reticuloendothelial cells had exuberant parasitic cytoplasmatic inclusions and a few images of erythrophagocytosis were present (Figure 1). The sample was sent to DNA PCR analysis and* Leishmania infantum* was identified. Previous bone marrow smears were reviewed and scarce parasites, not previously observed, were found in a smear performed 6 months after SCT. The donor had no history of visceral leishmaniasis. The patient was treated with a total dose of 24 mg/kg of liposomal amphotericin B, with clinical improvement and no signs of leishmania in the bone marrow. Five months later, the patient developed epigastric pain and duodenal biopsy revealed no evidence of graft versus host disease but the presence of inflammatory infiltrate with the presence of amastigotes in the macrophages was evident; a new bone marrow was performed and was positive for leishmania. The patient restarted liposomal amphotericin B and a prolonged maintenance treatment is planned.

## 3. Discussion

The classical presentation of visceral leishmaniasis includes fever, hepatoesplenomegalia, and cytopenias [1–5]. Visceral asymptomatic leishmaniasis is increasingly reported in patients living in endemic areas and in immunocompromised hosts [5–10]. This protozoon is endemic in the Mediterranean basin and patient reported sporadic contact with domestic dogs, which can be a reservoir. It is worth noting that dog leishmaniasis is reported to be common in the residence area of this patient. T-lymphocytes play a major role in the host defense from this parasitic infection and allogeneic bone marrow receipts under immunosuppression therapy are a particular susceptible population, especially in endemic areas [1]. There must be a high suspicion index evaluating this patients bone marrow samples as they can easily establish this diagnosis. This clinical case is remarkable for the absence of organomegaly, constitutional symptoms, and the massive invasion of bone marrow. We could not establish clearly the source of infection.
